# Sex and Breed-Dependent Organ Development and Metabolic Responses in Foetuses from Lean and Obese/Leptin Resistant Swine

**DOI:** 10.1371/journal.pone.0066728

**Published:** 2013-07-23

**Authors:** Laura Torres-Rovira, Anne Tarrade, Susana Astiz, Eve Mourier, Mariluz Perez-Solana, Paloma de la Cruz, Ernesto Gomez-Fidalgo, Raul Sanchez-Sanchez, Pascale Chavatte-Palmer, Antonio Gonzalez-Bulnes

**Affiliations:** 1 INIA, Madrid, Spain; 2 INRA, UMR1198 Biologie du développement et reproduction, Jouy-en-Josas, France; University of Sydney, Australia

## Abstract

The present study aimed to determine the effects of breed and sex on growth patterns and metabolic features of advanced-pregnancy foetuses exposed to the same environmental conditions. Thus, at Day 62 of pregnancy, swine foetuses from an obese breed with leptin resistance (Iberian breed) were compared to lean crossbred foetuses (25% Large White ×25% Landrace ×50% Pietrain). There were differential developmental patterns in foetuses with leptin resistance, mainly a higher relative weight of the brain resembling “brain-sparing effect”. Prioritization of brain growth may be protective for the adequate growth and postnatal survival of the Iberian individuals, an ancient breed reared in extensive semi-feral conditions for centuries. There were also clear sex-related differences in foetal development and metabolism in the Iberian breed. Female Iberian foetuses were similar in size and weight to male littermates but had a significantly higher relative liver to body weight ratio resembling “liver-sparing effect” and a trend for a higher relative intestine to body ratio. Moreover, the availability of triglycerides, cholesterol and IL-6 in female Iberian foetuses was similar to that of lean crossbred foetuses. Overall, these features may favour a better postnatal survival and development of females, the sex more critical for the species survival. These findings set the basis for future translational studies aimed at increasing the knowledge on the interaction between genetic and environmental factors in the early programming of the adult phenotype.

## Introduction

Obesity and obesity-associated hyperleptinaemia caused by *leptin resistance* have been linked to reproductive disorders in human beings [1], [2] and some animal species like the pig [3], [4]. In both obese human and pigs, poor reproductive outcomes have been related to impairments of ovarian function and, mainly, to deficiencies in endometrial receptivity, implantation and early embryo development [5]–[12].

In human pregnancies, foetal development may be afterwards hampered by either restricted- or excess-growth. Offspring will be born small- or large-for-gestational-age at birth, respectively, as early described in humans by Prof. Smith in the late 1940's [13], [14]. The most troubling fact is that, in both cases, life-long health and fitness of offspring are affected. Evidences of the determinant role of the prenatal environment on the adult phenotype, strongly reinforced by birth-records of the Dutch Hunger Winter [15]–[17] and the studies of Prof. Barker [18], gave way to the concept of the Developmental Origin of Health and Disease (DOHaD; [19]). Currently, the hypothesis of DOHaD is well-supported by abundance of data from different mammalian species [20].

In pigs, it is important to study the effects of prenatal nutrition on the adult phenotype both from the viewpoint of their use as biomedical models but also from the viewpoint of animal production, health and welfare [21]. The failure of some foetuses in the litter for achieving their genetically determined potential size and, thus, the birth of some piglets smaller than normal size (intrauterine growth retardation; IUGR) are common and widely described for lean swine. IUGR in lean pigs is mainly due to intrauterine crowding and subsequent placental insufficiency in high-prolific lines [21]–[23]. Information on the occurrence of IUGR in obese breeds is scarcer than in lean swine, but previous studies in obese Iberian pigs provide strong evidence of a high incidence of IUGR occurring from very early-pregnancy stages [10], [24], in spite of a low prolificacy. Piglets affected by IUGR have high rates of mortality and morbidity at early postnatal stages. The health and welfare of the surviving IUGR offspring are compromised by gastrointestinal, metabolic, respiratory and immune dysfunctions, and they have reduced growth potential and a high predisposition for adiposity [24]–[28], which bears a resemblance with findings in human medicine.

The concept of DOHaD states that the adult phenotype is the result of the interaction between prenatal nutrition and early postnatal environments, but there is also a strong influence of genetic background [29]. In this context, the availability of obese and lean swine breeds displaying IUGR provides the possibility of an interesting model for the study of the role of such genetic predisposition. The most representative obese breed is the Iberian pig (Sus scrofa meridionalis), which is genetically different from the modern commercial breeds (Sus scrofa ferus, [30], [31]). These animals have been reared in semi-feral conditions for centuries and have coped with seasonal cycles of feasting and famine by storing excess fat during food abundance [32], [33]. In fact, Iberian pigs have a higher voluntary food intake and a higher trend towards adiposity than lean swine breeds [34]–[36]. The abundance of fat increases secretion of leptin [37], which should diminish food intake. However, the Iberian pig has a gene polymorphism for the leptin receptor (LEPR), which has not been found in lean swine breeds, similar to the syndrome of leptin resistance described in human medicine [38]–[40]. The LEPR gene polymorphism of the Iberian breed has effects on food intake and fat deposition [35], [36] and, thus, Iberian pigs are prone to obesity. Hence, the Iberian swine, having in mind is unique characteristic of leptin resistance, is specially useful as a robust, amenable and reliable translational model for studies on obesity, metabolic syndrome and nutrition-associated diseases in humans [41], [42].

Thus, the aim of the present study was to set a basis for such studies by determining possible breed-related differences in growth patterns and metabolic features of the feto-placental unit at advanced pregnancy stages through the comparison of obese Iberian and lean commercial crossbred sows. Foetal development and viability are largely known to be affected by the litter size and, thus, availability of uterine space [43]. Hence, we used a commercial lean crossbred strain (25% Large White ×25% Landrace ×50% Pietrain) selected for meat efficiency but not for prolificacy to match the low prolificacy of the Iberian breed. At the same time, to avoid any influence from maternal nutrition, both breeds were fed with the same diet, adjusted to fulfil pregnancy requirements according to individual weights.

## Materials and Methods

### Ethics statement

The experiment was carried out under Project License from the INIA Scientific Ethic Committee and animal manipulations were performed according to the Spanish Policy for Animal Protection RD1201/05, which meets the European Union Directive 86/609 about the protection of animals used in research. We hereby confirm that the INIA Scientific Ethic Committee, which is the named IACUC for the INIA, specifically approved this study (report CEEA 2010/003).

### Animals and management

The study involved seven Iberian (group IB) and six lean crossbred sows (25% Large White ×25% Landrace ×50% Pietrain; group L) that became pregnant after cycle synchronization with altrenogest (Regumate®, MSD, AH, Boxmeer, The Netherlands) and insemination to purebred litters. All the females were nulliparous, were treated at the same time, and have twelve months of age when they were inseminated. The IB sows had been genotyped for polymorphism on *LEPR* gene with protocols previously described [35] and found to be homozygous for the allele LEPRc.1987T. All the females from both groups were housed together in collective pens at the facilities of the INIA Animal Laboratory Unit (Madrid, Spain), which meets the requirements of the European Union for Scientific Procedure Establishments. The sows were individually fed with a standard grain-based diet (89.8% of dry matter, 15.1% of crude protein, 2.8% of polyunsaturated fat and 3.00 Mcal/kg of metabolizable energy) with *ad libitum* access to water. The amount of food offered to each sows was adjusted to body-weight and calculated for fulfilling the requirements of pregnancy development, based on data from the British Society of Animal Science [44].

The experimental sampling was performed in all the sows at Day 62 of pregnancy.

### Morphometric evaluation and sampling of sows, genital tracts and foetuses

At Day 62 of pregnancy (around mid-pregnancy), all sows were weighed and measured for back-fat depth. Fat depth was determined by using a SonoSite S-Series ultrasound machine equipped with a multifrequency (5–8 MHz) lineal array probe (SonoSite Inc., Bothell, WA). The probe was placed against the skin, in a point at the right side of the animal located at 4 cm from the midline and transversal to the head of the last rib as determined by palpation. Concurrently, blood samples were drawn by puncture of the orbital sinus [45], into 5 ml sterile heparinized vacuum tubes (Vacutainer™ Systems Europe). Immediately after recovery, the blood was centrifuged at 1500 g for 15 min and the plasma was separated and stored into polypropylene vials at −20°C until assay for determination of leptin and parameters of carbohydrate, protein and lipids metabolism. Subsequently, the sows were euthanized by i.v. injection with a euthanasia solution (T-61, MSD AH, Boxmeer, The Netherlands). The entire genital tracts were immediately collected for morphometric examination, weighing and sampling.

Ovulatory sites in the ovaries were assessed for the determination of ovulation rate and for the evaluation of morphologically normal and regressing corpora lutea. Regressing corpora lutea were white opaque, smaller in size than normal corpora lutea and scarcely vascularised, as previously described [10]. Samples of two normal corpora lutea from each ovary were used for *in vitro* culture and determination of progesterone secretion.

Each uterus was measured in length and weighted. Afterwards, its content was exposed and the conceptuses were recorded according to their position. Immediately, for each conceptus, blood samples were drawn from heart and/or umbilical cord with heparinised syringes, samples of allantoic and amniotic fluids were obtained by aspiration through the chorioallantoic and amniochorion membranes, respectively, and one sample of around 1 cm^2^ of placental tissue (including trophoblast and uterine endometrium) was collected for histological evaluation. Allantoic and amniotic fluids were centrifuged at 1500 g for 15 min and supernatants were stored into polypropylene vials at −20°C until assay. Samples of placental tissue were immediately fixed with 4% paraformaldehyde. Finally, all the conceptuses were recovered to be sexed, measured and weighted and the empty uterus was weighted.

### Culture of luteal tissue and assessment of progesterone secretion

The technique for *in vitro* culture of luteal tissue was adapted from procedures previously described [46]. Briefly, each corpus luteum was sliced into small strips (each one weighing around 40 mg) and washed several times in sterile saline solution. Two individual strips from each corpus luteum were placed in culture glass tubes (one strip/tube) with culture medium (Dulbecco's Modified Eagle's Medium and Ham's F-12 medium 1∶1 [v.v]; Sigma-Aldrich Chemie, Steinheim, Germany) and one of the tubes was supplemented with 100 ng/ml of porcine LH (bio-potency 428 UI/mg; Laboratorios Calier, S.A, Barcelona, Spain). The strips were then incubated, on a shaking platform, for 24 hours at 38.5°C and 5% CO_2_ atmosphere. After incubation, the media was recovered and frozen at −20°C until measurement of progesterone. The strips were blotted and weighed, in order to determine the hormone secretion in terms of ng per mg of luteal tissue.

Progesterone concentrations in plasma and culture media were measured in a single analysis using enzymeimmunoassay kits (Demeditec Diagnostics GmbH, Kiel-Wellsee, Germany). The assay sensitivity was 0.04 ng/ml and the intra-assay variation coefficient was 5.4%. Subsequently, the percentage of LH-stimulated strips for which progesterone secretion was more than 5% of the basal production of their untreated counterparts (positive reactivity) was also assessed.

### Evaluation of absolute and relative measures and weights of the foetuses

Immediately after recovery, crown-rump length, thoracic diameter, occipito-nasal length and biparietal diameter were measured in all the foetuses. The total weight of the foetus and the weights of the head, trunk and main organs (brain, heart, liver, intestine and kidneys) were also assessed. Afterwards, the following weight ratios were considered: weight of the uterus relative to the individual and total foetal weight, weight of the foetal head relative to total weight and trunk weight and brain weight relative to the weight of total viscera, heart, liver, kidneys and intestine.

### Evaluation of maternal and foetal metabolic status

Concentrations of leptin in maternal plasma were determined in a single analysis using the Multi-species Leptin RIA kit (Demeditec Diagnostics GmbH, Kiel-Wellsee, Germany). The assay sensitivity was 1.0 ng/ml; the intra-assay variation coefficient was 3.1%.

Parameters related to the metabolism of glucose and lipids (triglycerides, total cholesterol, high-density lipoproteins cholesterol [HDL-c] and low-density lipoproteins cholesterol [LDL-c]) were measured in maternal and foetal plasma and allantoic and amniotic fluids with a clinical chemistry analyzer (Screen Point, Hospitex Diagnostics, Sesto Fiorentino, Italy), according to the manufacturer's instructions.

### Evaluation of foetal endocrine and inflammatory status

Concentrations of oestradiol and IL-6 in foetal plasma and allantoic and amniotic fluids were measured by using enzymeimmunoassay kits (Demeditec Diagnostics GmbH, Kiel-Wellsee, Germany for estradiol and DuoSet ELISA Development kit from R&D Systems Europe, Ltd. Abingdon, UK for IL-6). The assay sensitivities were 1.4 pg/ml and 100 pg/ml for oestradiol and IL-6, respectively; intra-assay variation coefficients were 5.7% for estradiol and 6.8% for IL-6, respectively.

### Evaluation of placental histology

Samples of placental tissue were maintained in 4% paraformaldehyde for three hours at room temperature and overnight at 4°C. They were transferred to PBS the next day and maintained at 4°C until processed for histology and paraffin embedded. Sections of 7 µm were cut and subsequently stained with haematoxylin-eosin, mounted and scanned (Nanozoomer Digital Pathology 2.3, Hamamatsu Photonics, Hamamatsu, Japan). Stereology was performed on each section using the One Stop Stereology technique of Mercator software (Explora Nova, La Rochelle, France) for determining volume fractions of foetal vessels, connective tissue, trophoblast, uterine glands and maternal vessels, as described [47].

### Statistical analyses

Effects of breed on metabolic and reproductive features of the sows and effects of the breed, sex and normal/IUGR status of the foetus on morphological, endocrine, metabolic and inflammatory characteristics of the conceptuses were assessed by analysis of variance (one-way ANOVA) or by a Kruskall–Wallis test when a Levene's test showed non-homogeneous variables. IUGR was defined as the observation of an individual weight under one standard deviation of the mean litter value, as described elsewhere [15]. All the results were expressed as mean ± SEM and statistical significance was accepted from *P*<0.05.

## Results

### Effect of breed on maternal fatness, metabolism and reproductive features

Weight, subcutaneous fat depth and plasma concentrations of lipid metabolites were significantly different between Iberian (group IB) and lean crossbred sows (group L). The IB females were lighter (131.1±2.6 vs. 164.7±9.5 kg, *P* = 0.01), but had more back-fat depth (36.9±0.7 vs. 16.7±1.5 mm, *P* = 0.0005). Plasma concentrations of leptin (2.6±0.4 vs. 1.8±0.1 ng/ml, *P* = 0.08), triglycerides (56.6±8.1 vs. 27.7±7.9 mg/dl, *P* = 0.02) and cholesterol (94.3±5.3 vs. 72.8±4.3 mg/dl, *P* = 0.01; mainly, LDL-c: 57.9±4.7 vs 44.9±5.4 mg/dl, *P* = 0.09) were significantly higher in IB sows.

The mean number of ovulatory sites on the ovaries was significantly lower in the IB compared to the L group (14.4±0.7 vs. 22.5±1.4, *P* = 0.0005). The same was found when comparing only corpora lutea with adequate development (10.7±0.4 vs. 15.5±1.5, *P* = 0.007). The number of viable foetuses, however, was not different between the two breeds (7.1±1.2 in the IB vs. 8.2±1.6 in the L group, *P* = 0.623).

Plasma progesterone concentrations were significantly lower in IB compared to lean sows (37.9±2.6 vs. 64.5±3.6 ng/ml, *P*<0.005). Similarly, both basal and LH-induced *in vitro* secretion of progesterone were significantly lower in IB than in L females (39.9±3.3 vs. 70.7±5.3 and 52.5±5.7 vs. 76.7±6.4 ng/mg of luteal tissue, *P*<0.0001 for both).

### Effect of breed and sex on foetal development

#### General observations

The data obtained from the evaluation of a total of 50 IB and 49 L foetuses (around 50% of males and females in both groups) are detailed in Table 1. Foetuses from the L group were 11% larger and 20% heavier than IB foetuses (*P* = 0.0001 for both variables). In contrast, the total foetal weight to total uterine weight ratio was higher in IB foetuses (21.2±1.7 vs 15.5±1.2; *P* = 0.005).

**Table 1:** Effects of breed and sex on foetal development.

	Iberian breed		Lean crossbred	
	Female	Male	Female	Male
**Body length (cm)**	10.3±0.1^i^		11.5±0.1^j^	
	10.2±0.1^i^	10.3±0.2^i^	11.4±0.2^j^	11.6±0.1^j^
**Occipito-nasal length (cm)**	4.9±0.1^e^		5.1±0.1^f^	
	4.9±0.1^a^	4.9±0.1^a^	5.1±0.1^b^	5.1±0.1^b^
**Biparietal diameter (cm)**	2.5±0.1^i^		2.7±0.1^j^	
	2.5±0.1^e^	2.6±0.1^c^	2.8±0.1^f^	2.7±0.1^d^
**Trunk diameter (cm)**	4.0±0.1^i^		4.3±0.1^j^	
	4.0±0.1^a^	4.0±0.1^e^	4.2±0.1^b^	4.3±0.1^f^
**Body weight (g)**	122.8±2.2^i^		152.8±2.5^j^	
	119.0±2.7^i^	126.0±3.3^i^	146.0±3.2^ j,3^	159.4±3.4^j,4^
**Head weight (g)**	30.4±0.5^i^		34.9±0.5^j^	
	30.4±0.6^i^	30.6±0.8^i^	33.8±0.6^j ,1^	36.0±0.8^ j,2^
**Trunk weight (g)**	90.6±1.8^i^		116.2±2.1^j^	
	87.9±2.2^i^	93.5±2.7^i^	110.4±2.7^j,3^	121.7±2.9^j,4^
**Brain weight (g)**	4.2±0.1^a^		4.0±0.1^b^	
	4.2±0.1^c^	4.3±0.1	3.8±0.1^d,1^	4.1±0.1^2^
**Heart weight (g)**	0.8±0.1^i^		1.1±0.1^j^	
	0.7±0.1^i^	0.7±0.1^i^	1.1±0.1^j^	1.2±0.1^j^
**Liver weight (g)**	6.1±0.2^i^		7.4±0.2^j^	
	6.2±0.2^a^	6.0±0.2^i^	6.9±0.2^b,1^	7.7±0.2^ j, 2^
**Intestine weight (g)**	3.0±0.1^e^		3.4±0.1^f^	
	3.1±0.1	2.9±0.2^e^	3.3±0.1	3.6±0.2^f^
**Kidneys weight (g)**	1.4±0.1^i^		2.3±0.1^j^	
	1.4±0.1^i^	1.4±0.1^i^	2.2±0.1^j^	2.4±0.1^j^

Mean ± S.E.M. measurements and weights of Iberian purebred and lean crossbreed foetuses and selected organs.

Different letter in superscripts indicate significant differences between breeds (a≠b, p<0.05; c≠d, p<0.01; e≠f, p<0.005; g≠h, p<0.001; i≠j, p<0.0005) whilst different number denotes significant differences between sexes within the same breed (1≠2, p<0.05; 3≠4, p<0.01; 5≠6, p<0.005).

#### Organ weights in all foetuses

**Figure 1:**
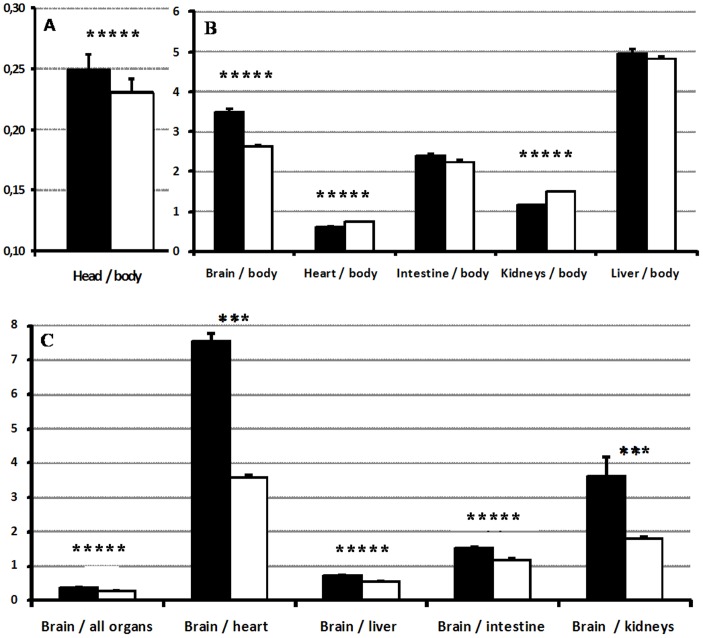
Effects of breed on the relative development of foetuses and their organs. Mean ± S.E.M. ratios of head weight to total body weight (A), of individual weight of selected organs to total body weight (B) and of brain weight to the weights of total viscera and selected organs (C) in Iberian purebred (black bars) and lean crossbreed foetuses (white bars). Asterisk indicate significant differences between breeds (* = p<0.05, ** = p<0.01, *** = p<0.005, **** = p<0.001, ***** = p<0.0005).

Individual organs were also significantly heavier in L foetuses (Table 1). However, the relative weight of the head and brain compared to total body weight (Figure 1, *P* = 0.0005 for both) and to other organs (Figure 1, *P*<0.005 for all) was significantly higher in IB foetuses. The relative weight of intestine/body also tended to be higher in IB foetuses (*P* = 0.05), whilst the ratios of heart and kidneys weights to body weight were higher in the L foetuses (*P* = 0.0005 for both).

#### IUGR foetuses

IUGR was found in 16.7% of the L and 21.9% of the IB foetuses. Relative brain to body weight, weight of total thoracic viscerae, of total abdominal viscerae, of liver (*P*<0.005 for all) and weight of intestine (*P* = 0.04) was significantly higher in the IB foetuses affected by IUGR when compared to littermates with adequate development, but this was not the case in the L conceptuses with IUGR.

#### Sex differences

In the L group, male and female foetuses had similar sizes but male foetuses were 9% heavier (Table 1, *P* = 0.006). There were no significant sex-related differences in the IB group. There were no sex-related effects on the head and brain to entire body and organs ratios in any breed. In the IB foetuses, however, the liver to body weight ratio was higher in females compared to males (*P* = 0.04). There was also a trend (*P* = 0.06) for a higher ratio of intestine to body weight in females than males.

### Effect of breed, sex and IUGR on foetal endocrine, metabolic and inflammatory features

Oestradiol concentrations were significantly higher in the amniotic fluids of IB conceptuses (266.3±31.0 vs. 142.6±12.9 pg/ml in the L group, *P* = 0.002); without differences related to IUGR or sex.

Parameters related to glucose and lipid metabolism in foetal plasma are described in Tables 2 and 3. Plasma glucose and cholesterol concentrations, mainly LDL-c, were significantly higher in IB compared to L conceptuses (*P*<0.01 for all), due to significantly higher values in IB males than in L males for total cholesterol and LDL cholesterol (*P*<0.005) and for triglycerides (*P* = 0.06). On the other hand, triglyceride concentrations were lower in the IB foetuses (*P* = 0.009), without differences between sex. Amniotic fluid concentrations were consistently lower in IB compared to L foetuses (*P*<0.05 for all) whereas only total and LDL cholesterol were found to be lower in IB compared to L groups for allantoic fluid (*P* = 0.06 and *P* = 0.006, respectively).

**Table 2:** Effects of breed and sex on foetal glucose metabolism.

	Iberian breed		Lean crossbred	
	Female	Male	Female	Male
**Glucose in foetal plasma (mg/dl)**	52.6±4.1^i^		24.5±3.7^j^	
	55.0±4.6^i^	50.9±6.4^e^	25.6±3.7^j^	23.1±2.7^f^
**Glucose in amniotic fluid (mg/dl)**	32.8±1.7^ a^		39.1±2.4^ b^	
	34.2±2.8	31.1±1.8	40.1±3.6	38.2±3.2
**Glucose in allantoic fluid (mg/dl)**	14.6±1.9		13.3±1.4	
	17.1±2.8	11.7±2.3	14.1±1.9	12.6±1.9

Mean ± S.E.M. glucose concentrations in plasma and allantoic and amniotic fluids of Iberian purebred and lean crossbreed foetuses.

Different letter in superscripts indicate significant differences between breeds (a≠b, p<0.05; c≠d, p<0.01; e≠f, p<0.005; g≠h, p<0.001; i≠j, p<0.0005).

**Table 3:** Effect of breed and sex on parameters related to lipids metabolism.

	Iberian breed		Lean crossbred	
	Female	Male	Female	Male
**Triglycerides in foetal plasma (mg/dl)**	38.1±1.7^c^		46.3±2.5^d^	
	39.9±2.0	36.7±2.6	46.7±3.6	45.7±3.5
**Triglycerides in amniotic fluid (mg/dl)**	3.2±0.2^a^		4.5±0.4^b^	
	3.0±0.4^a^	3.5±0.3	4.3±0.6^b^	4.6±0.6
**Triglycerides in allantoic fluid (mg/dl)**	9.5±1.3^a^		14.1±2.2^b^	
	8.7±1.2	10.6±2.4	11.5±2.5	16.5±3.5
**Total cholesterol in foetal plasma (mg/dl)**	66.5±2.4^c^		56.2±2.4^d^	
	64.3±3.5	68.2±3.1^e^	60.0±3.4	51.6±3.0^f^
**Total cholesterol in amniotic fluid (mg/dl)**	6.2±0.3		7.6±0.7	
	6.0±0.5	6.4±0.5	6.7±0.8	8.4±1.0
**Total cholesterol in allantoic fluid (mg/dl)**	5.6±0.5		8.4±1.4	
	5.7±0.8	5.4±0.6	7.2±1.6	9.4±2.2
**HDL-c in foetal plasma (mg/dl)**	7.7±0.6		6.2±0.9	
	6.2±1.0^1^	8.9±0.7^a,2^	6.7±1.4	5.6±1.2^b^
**HDL-c in amniotic fluid (mg/dl)**	1.9±0.2		1.9±0.4	
	1.6±0.3	2.2±0.4	1.6±0.6	2.1±0.6
**HDL-c in allantoic fluid (mg/dl)**	2.9±0.6		5.5±2.0	
	3.4±0.9	2.1±0.9	7.2±2.9^1^	2.9±1.8^2^
**LDL-c in foetal plasma (mg/dl)**	51.2±2.1^c^		42.1±2.1^d^	
	50.2±3.0	52.0±3.0^e^	45.3±3.2	38.1±2.4^f^
**LDL-c in amniotic fluid (mg/dl)**	4.6±0.3^a^		6.1±0.6^b^	
	4.5±0.5	4.7±0.4	5.8±0.8	6.4±0.9
**LDL-c in allantoic fluid (mg/dl)**	3.7±0.4^c^		6.7±1.0^d^	
	3.4±0.5^c^	4.7±0.7^a^	8.5±1.6^d^	8.5±1.7^b^

Mean ± S.E.M. concentrations of triglycerides, total cholesterol, high-density lipoproteins cholesterol [HDL-c] and low-density lipoproteins cholesterol [LDL-c] in plasma and allantoic and amniotic fluids of Iberian purebred and lean crossbreed foetuses.

Different letter in superscripts indicate significant differences between breeds (a≠b, p<0.05; c≠d, p<0.01; e≠f, p<0.005; g≠h, p<0.001; i≠j, p<0.0005). Different number in superscripts indicate significant differences between sexes within the same breed (1≠2, p<0.05; 3≠4, p<0.01; 5≠6, p<0.005).

The comparison between foetuses with adequate development and foetuses affected by IUGR showed no significant differences in the IB breed, but IUGR conceptuses from the L group showed a trend for a higher plasma cholesterol concentrations (66.7±11.0 vs. 54.7 2.2, *P* = 0.09), due to a significant higher level of HDL-c (14.4±2.2 vs. 5.0±0.7, *P*<0.0005).

IL-6 concentrations in the foetal plasma and allantoic and amniotic fluids were significantly lower in IB than in L conceptuses (3046.9±489.7 vs. 5531.6±560.4 pg/ml, *P* = 0.001). Moreover, IL-6 concentration was significantly lower in IB than in L males (2746.3±488.3 vs. 6569.8±694.5 pg/ml, *P*<0.0005) but not in females. IL-6 concentrations were also significantly lower in IUGR compared to normal foetuses of both breeds (*P*<0.05).

### Effect of breed, sex and IUGR on placental features

There were no significant differences in the stereological analysis of the placental components among breeds, sexes and normal/IUGR foetuses, except for a higher volume fraction of trophoblastic vessels in the IB IUGR foetuses when compared to normal littermates (10.9±1.3 vs. 7.6±0.6, *P* = 0.02), with no difference between males and females.

## Discussion

The present study evidences the existence of differential developmental patterns in Iberian foetuses, which would be protective for the adequate growth and postnatal survival of the individuals. Such differential patterns were more obvious in female compared to male foetuses which, in addition to sex-related differences in the hypothalamic expression of anorexigenic and orexigenic peptides at birth (Ovilo, unpublished data), would explain the early-postnatal catch-up growth previously reported in female, rather than male, piglets of the Iberian breed [24].

The analysis of maternal phenotypes in the present study clearly confirmed the trend for the Iberian pig for a higher adiposity than modern commercial lean crosses, despite the fact that they ate the same maintenance diet. The Iberian sows of the current trial were fatter and had higher plasma concentrations of leptin, triglycerides and cholesterol than their lean crossbred counterparts. The results of the present study also indicate a lower ovulatory quota for the Iberian breed when compared to the lean females, in agreement with some previous studies [10] but opposite to other results obtained by our group [48], [49]. The inconsistencies among these studies may be explained by the results of a subsequent trial which highlighted differences in ovulatory patterns between different Iberian dam-lines and their critical importance when studying prolificacy in the Iberian breed [12]. The data of the present trial also evidence a lower progesterone secretion in the Iberian than in the lean sows, supporting previous findings obtained when comparing Iberian and lean crosses [46]. Overall, the results of the current study emphasize previous information suggesting that the main factor determining prolificacy in both Iberian and lean crossbred swine is embryo/foetal viability [10], [12], [23], [50].

The analysis of foetal phenotypes in the present study clearly indicates breed-related differences in weight and size between foetuses of Iberian and lean-crossbred sows. Such differences may be explained by intrinsic differences in body size and weight between both groups rather than by a reduced developmental rate of Iberian foetuses. Indeed, Iberian pigs are significantly smaller than commercial lean pigs, even in neonatal stages [51].

Apart from breed-related differences in body weight and size, a crucial result of the present study is the existence of a very clearly different developmental pattern in Iberian and lean-crossbred conceptuses. Organ analysis indicates that the relative weight of the brain was higher in the Iberian foetuses, whilst the relative weights of other organs like heart and kidneys were lower, when compared to lean counterparts. These data suggest that brain growth is prioritized in the Iberian foetuses and resemble the “brain-sparing effect”, first described in humans [52]. The brain-sparing effect has been associated in many mammals to intrauterine growth retardation (IUGR) by nutritional and metabolic deficiencies [21], [53], [54]. In the current study, there were no significant differences in the occurrence of IUGR between groups. However, similarly to the normal foetuses, the brain-sparing effect was more evident in the Iberian IUGR foetuses. The objective of brain-sparing is to assure that brain growth is protected, since a failure in the supply of oxygen and/or nutrients to the developing brain causes a broad spectrum of adverse neurological outcomes [55], [56]. Such deficiencies can compromise the critical functions such as breathing, suckling and/or any of the so called autonomic functions [57]. Hence, brain sparing assures vitality and survival of the neonate.

The relatively more advanced growth of the brain in the Iberian foetuses suggests the existence of a strong protective mechanism to improve postnatal survival of Iberian piglets. The main factors determining optimal brain development are genetics and environment [58], [59]. The Iberian swine has an ancient origin, being traced back approximately to year 1000 BC. It has been scarcely selected and has been reared in extensive semi-feral conditions for centuries. In such conditions, the survival of the piglets is largely dependent on their vitality, vivacity and ability for suckling, moving and interacting with their mother. We can conjecture that the brain-sparing characteristics observed in Iberian piglets may support these features and, hence, may favour a better postnatal survival.

The occurrence of breed-related protective effects is also supported in our study by the finding of a significant higher volume fraction of foetal vessels in the Iberian IUGR foetuses when compared to normal littermates; such difference was not found in the lean foetuses. This increase in foetal vessels may be a compensatory response to improve the blood flow and the efficiency of nutrients transport. These data are consistent with those of Blomberg [60] which demonstrated an increase of the expression of endothelial-constitutive nitric oxide synthases (eNOS or NOS3), implicated in blood vessel dilatation or growth, in the IUGR placenta at the same period. The nitric oxide (NO) pathway for increasing angiogenesis and adequate development of embryo blood flow is associated with the secretion of estrogens by the conceptus [61], which, concurrently, was found to be higher in the Iberian foetuses of the current trial.

The other main result of the present study is the existence of sex-related effects in developmental patterns of the Iberian foetuses, which were not found in the lean crossbred conceptuses. The males were significantly heavier than the female foetuses in the lean group whilst the weight of male and female Iberian foetuses was similar, which indicates an enhanced development of the females in this breed. As a matter of fact, male and female Iberian offspring have similar weights and body measurements at birth. In case of critical maternal malnutrition, however, newborn females are lighter but develop early-postnatal catch-up growth to reach higher body weights than their brothers [24]. Such sex-related catch-up growth would be favoured by a lower hypothalamic expression of anorexigenic peptides and a higher expression of orexigenic ones (Ovilo, unpublished results) and possibly by faster intestinal development and improved food absorption, since the Iberian female foetuses showed a trend for higher intestine to body weight ratios than their male counterparts. Advanced development of the intestine improves neonatal absorption and utilization of nutrients and other substances like immunoglobulins and, thus, would increase the survival possibilities and the performance of the piglet [62].

Furthermore, the relative liver to body weight ratio was higher in Iberian female foetuses compared to males. Faster liver development may favour postnatal growth [63], through a “liver-sparing effect”, which appears in addition to the “brain-sparing effect” in foetuses undergoing adverse conditions [64]. The equilibrium or the predominance of one or another effect is driven by the availability of nutrients and even by the foetal sex [65].

Adequate liver development is associated with an increase in hepatic glycogen deposition and activity of key gluconeogenic enzymes, thus enhancing hepatic glucogenic capacity in preparation for the nutritional transition from continuous maternal supply of nutrients via the placenta to a more intermittent supply from the milk via the intestine after birth [66]–[68]. Differences in foetal liver developmental patterns may interfere in the pre- and postnatal metabolism of nutrients other than glucose, such as amino-acids, proteins, lipids, vitamins and minerals [69]. The foetal liver is also actively participating in the synthesis of complex lipids (mainly cholesterol, [70]) that are essential for pre- and postnatal development [71]. In this way, foetal development is highly dependent on the adequacy of the maternal supply of cholesterol and triglycerides and foetal growth may be disturbed either by deficiency [54], [72]–[74] or excess [75], [76]. In the current study, the availability of triglycerides and cholesterol in female Iberian foetuses was similar to that of lean crossbred foetuses. Moreover, the availability of IL-6 was lower in Iberian males but similar in female Iberian and lean crossbred foetuses. Since IL-6 is a pleiotropic cytokine that it is also involved in glucose and lipid metabolism [77]–[79], these data strengthen the evidences of sex-driven differences in metabolism. More work is needed to understand the physiological implications of such differences.

Overall, all the features described above indicate a better postnatal survival and development of females, which are more critical for the survival of the species. Thus, these results support previous evidences in obese humans and murine models exposed to malnutrition indicating that female survival is higher compared to males [80]–[84].

In conclusion, the current study is supporting the existence of sex- and breed-related differences in morphometric, metabolic and inflammatory patterns in swine foetuses of obese and lean phenotypes that may be extended to postnatal stages. These findings set the basis for future translational studies aimed at increasing the knowledge on the interaction between genetic and environmental factors in the determination of adult phenotype [85]. Such studies are of paramount importance for understanding and preventing the rise of obesity and associated diabetes and cardiovascular diseases in populations adapted for surviving in harsh environments but currently exposed to nutrient excess, like China, India and Middle East countries [86].
